# Effects of chronic noise on the corticotropin-releasing factor system in the rat hippocampus: relevance to Alzheimer’s disease-like tau hyperphosphorylation

**DOI:** 10.1186/s12199-017-0686-8

**Published:** 2017-12-11

**Authors:** Zhihui Gai, Donghong Su, Yawen Wang, Wenlong Li, Bo Cui, Kang Li, Xiaojun She, Rui Wang

**Affiliations:** 1 Department of Occupational Hygiene, Tianjin Institute of Health and Environmental Medicine, Tianjin, 300050 China; 2 Shengli Oil Field Central Hospital, Dongying, 257034 China; 3 Shandong Academy of Occupational Health and Occupational Medicine, 18877, Jingshi Road, Lixia District, Jinan, 250062 China; 4 School of Public Health and Management, Weifang Medical University, Weifang, China; 5 Department of Occupational Hygiene, Institute of Health and Environmental Medicine, Academy of Military Medical Sciences, 1, Dali Road, Heping District, Tianjin, 300050 China

**Keywords:** Noise, Hippocampus, Corticotropin-releasing factor receptors, Phosphorylated tau

## Abstract

**Background:**

Chronic noise exposure has been associated with tau hyperphosphorylation and Alzheimer’s disease (AD)-like pathological changes, but the underlying mechanism is unknown. In this study, we explored the effects of long-term noise exposure on the corticotropin-releasing factor (CRF) system in the hippocampus and its role in noise-induced tau phosphorylation.

**Methods:**

Sixty-four rats were randomly divided into the noise-exposed group and the control group, and rats in the exposure group were exposed to 95 dB SPL white noise for 30 consecutive days. The levels of CRF, CRFR1, CRFR2, and total tau and phosphorylated tau (p-tau) at Ser396 (S396) and Thr205 (T205) in the hippocampus were measured at different time points after the final noise exposure. The co-localized distribution of CRF and p-tau (T205) in the hippocampus was evaluated using double-labeling immunofluorescence.

**Results:**

Long-term exposure to noise for 30 consecutive days significantly increased the expression of CRF and CRFR1 and their mRNAs levels in the hippocampus, which persisted for 7 days after final exposure. In contrast, CRFR2 was raised for 3–7 days following the last exposure. These alterations were also concomitant with the phosphorylation of tau at S396 and T205. Furthermore, there was co-localization of p-tau and CRF in hippocampal neurons.

**Conclusion:**

Chronic noise leads to long-lasting increases in the hippocampal CRF system and the hyperphosphorylation of tau in the hippocampus. Our results also provide evidence for the involvement of the CRF system in noise-induced AD-like neurodegeneration.

## Background

There are a great variety of adverse health effects induced by chronic environmental noise, including annoyance, hearing loss, cognitive impairment, cardiovascular disease, and increased risk of diabetes [1–3]. A number of reports have focused on the AD-like neuropathology, especially hyperphosphorylation of tau protein in the rodent hippocampus, due to the acute or chronic noise exposure [4, 5]. However, the underlying mechanisms of noise exposure to AD-like tau hyperphosphorylation in the hippocampus, a key structure in cognition and the initial area of tau pathology in AD [6], are still not well understood.

The corticotropin-releasing factor (CRF) signaling system plays a well-established role in triggering stress responses and acts as a general mediator/integrator of stress adaptations, whose receptors, CRF receptor 1 (CRFR1) and CRFR2, exert convergent biological effects on stress-related endpoints [7]. The dysregulation of the CRF signaling system in the hippocampus causes an AD-like pathology in animals [8–10]. Supporting a role for CRF in AD neuropathology, work from many laboratories has demonstrated that both CRF overexpression and acute or repeated exposure to stressors induce phosphorylation of tau and accumulation of Aβ within the hippocampus, a process that is dependent on CRFR1 [11, 12]. However, only limited information is available on the control of this system in the hippocampus under chronic noise stress.

In this study, we have investigated the effects of long-term noise exposure on the CRF signaling system and explored the relationship between CRF and noise-induced AD-like changes in the rat hippocampus. Our data could implicate CRF-dependent mechanisms in the neuropathophysiology induced by environmental noise.

## Methods

### Animals and experimental groups

**Fig. 1:**
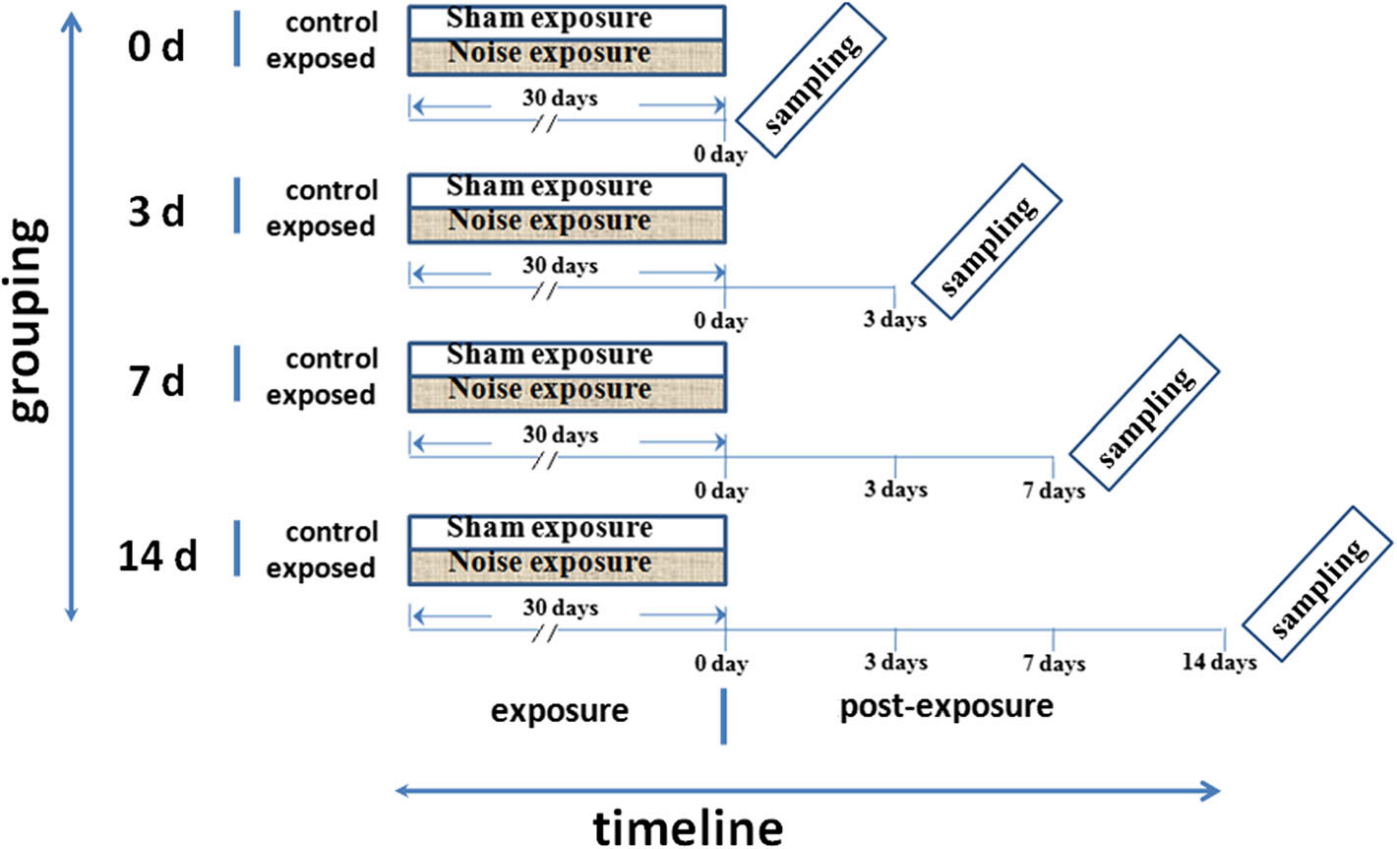
Experimental timeline. The rats were randomly assigned to four groups according to when the end-point evaluation was performed (0, 3, 7, and 14 days following the final noise exposure). Each group was further subdivided into control and exposed subgroups, in which the animals were subjected to 30 successive days of noise exposure as indicated by the gray area within the 30-day period. Sham (no noise) exposure, as indicated by the blank segments in the 30-day period, was performed on the animals in the control group for 30 days

Male Wistar rats (weighing 200–220 g, Lab Animal Center, Tianjin Institute of Health and Environmental Medicine, Tianjin, China) were used in the present study. All experimental procedures were performed in accordance with the guidelines approved by the Animal and Human Use in Research Committee of the Tianjin Institute of Health and Environmental Medicine. The rats were randomly divided into the noise-exposed group and the control group (32 rats per group). At different time points (days 0, 3, 7, and 14) after the final exposure, all rats in the study were sacrificed under brief anesthesia of chloral hydrate (30–35 mg/100 g) for subsequent biochemical analyses (Fig. 1).

### Noise exposure setup

All exposures were performed as described in our previous study [4]. Briefly, noise was generated using a noise generator and amplified with a power amplifier and delivered through a loudspeaker. Rats in the noise-exposed group were exposed to 95 dB SPL white noise for 30 consecutive days (4 h per day, from 8:00 to 12:00). Simultaneously, the control animals were housed in similar cages but only exposed to environmental background noise which was below 40 dB SPL.

### Determination of gene expression by real-time PCR

The hippocampi from noise-exposed and control rats were removed after the animals were sacrificed under chloral hydrate anesthesia at the time points indicated in the section “Animals and experimental groups” and homogenized by using oscillating mashers. RT-PCR was conducted as previously described by our laboratory [13]. Briefly, total RNA from tissues was extracted and reverse transcribed to cDNA successively. The primers and probes were designed for specific amplification of GAPDH (internal control gene), CRF, CRFR1, and CRFR2 sequences as shown in Table 1. The levels of *CRF* and *CRFR1/2* gene expression were examined by quantitative RT-PCR performed under the following cycling conditions: 2 min at 50 °C, 10 min at 95 °C, and then 45 cycles of 95 °C for 5 s followed by 57 °C for 30 s. Target gene levels of *CRF* and *CRFR1/2* were assessed after normalizing cycle thresholds (*C* = Ct_*CRF/CRFR1/CRFR2*_ − Ct_*GAPDH*_, and ΔΔCt = ΔCt_exposure_ − ΔCt_control_) relative to those of control group.

**Table 1:** Primers used for real-time RT-PCR of rat genes

Gene	Primers
CRF	F: 5′-CGCCCATCTCTCTGGATCT-3′
	R: 5′-TCTCCATCAGTTTCCTGTTGC-3′
CRFR1	F: 5′-GAACCTCATCTCGGCTTTCA-3′
	R: 5′-GGCTGTCACCAACCTACACC-3′
CRFR2	F: 5′-TCATCCTCGTGCTCCTCATC-3′
	R: 5′-GCCTTCACTGCCTTCCTGTA-3′
GAPDH	F: 5′-CAGGGCTGCCTTCTCTTGTG-3′
	F: 5′-GATGGTGATGGGTTTCCCGT-3′

*CRF*, corticotropin-releasing factor; *CRFR1*, corticotropin-releasing factor receptor 1; *CRFR2*, corticotropin-releasing factor receptor 2; *GAPDH*, glyceraldehyde-3-phosphate dehydrogenase

### Western blotting

The hippocampus preparation and Western blotting was conducted as described previously by our previous work [13]. Briefly, the tissue was dissected and homogenized in ice-cold radioimmunoprecipitation assay buffer. Homogenates were spun at 14,000×*g* for 15 min at 4 °C, and the supernatants were collected. Later, 20 μg/lane of sample was run on 10% sodium dodecyl sulfate polyacrylamide gels and transferred to microporous polyvinylidene fluoride membranes (0.45 μm, F. Hoffmann-La Roche Ltd., Germany). Immunoblots were probed with the specific antibodies as shown in the section “Primary antibodies,” and then incubated with peroxidase-conjugated species-specific anti-IgG secondary antibodies. After visualizing by enhanced chemiluminescence (EMD Millipore Co., USA), the intensity values of the immunoreactive signals were quantified by using Gel-Pro 3.1 software (Media Cybernetics Inc., USA).

### Double-labeling immunofluorescence microscopy

After perfusion by using 4% paraformaldehyde through the heart, the brain tissues were post-fixed in 4% paraformaldehyde, immersion fixed for 72 h, paraffin embedded, and then sectioned at 6 μm. After washing in TBS, sections were incubated for 30 min in 0.5% Triton X-100 to permeabilize the tissue. Sections were blocked with 10% goat serum in TBS for 2 h at 37 °C to reduce nonspecific binding and then were incubated for 1 h at 37 °C with anti-CRF and anti-p-tau (T205) antibodies diluted in TBST with 10% goat serum before incubating overnight at 4 °C. The following day, the sections were washed and incubated with FITC-labeled anti-goat antibody (1:200, ZSGB-BIO) and rhodamine-conjugated anti-rabbit antibody (1200, ZSGB-BIO), diluted in TBS with 10% goat serum, for 1 h at 37 °C. After washing, the sections were coverslipped with Antifade Mounting Medium and examined using a fluorescence microscope (Olympus DP71, Japan).

### Primary antibodies

Affinity-purified rabbit anti-CRF (polyclonal, 1:1000; Bioworld Technology, USA), anti-CRFR1 (polyclonal, 1:1500; Bioworld Technology, USA), anti-CRFR2 (polyclonal, 1:1500; Bioworld Technology), anti-TauC specific to C-terminal epitope of tau independent of its phosphorylation (polyclonal, 1:500, Santa Cruz Biotechnology, USA), anti-p-tau specific to p-T205 (polyclonal, 1:500, Bioworld Technology), anti-p-tau specific to p-S396 (polyclonal, 1:500, Santa Cruz Biotechnology, USA), and anti-GAPDH (1:10,000; Bioworld Technology) antibodies were used to detect hippocampal protein levels by Western blotting. Goat anti-CRF (polyclonal, 1:500; Santa Cruz Biotechnology, USA) and rabbit anti-p-tau (T205) (polyclonal, 1:400; Bioworld Technology) were used to label CRF and p-tau in the double-labeling immunofluorescence assays.

### Statistics

The data presented in the figures show group means ± SD unless otherwise noted. All data were processed by Student’s *t* tests using SPSS 19.0 software (SPSS, Inc., USA). Statistical significance levels were set at *p* < 0.05 for all tests.

## Results

### Chronic noise exposure causes abnormalities of the CRF system in the hippocampus

**Fig. 2:**
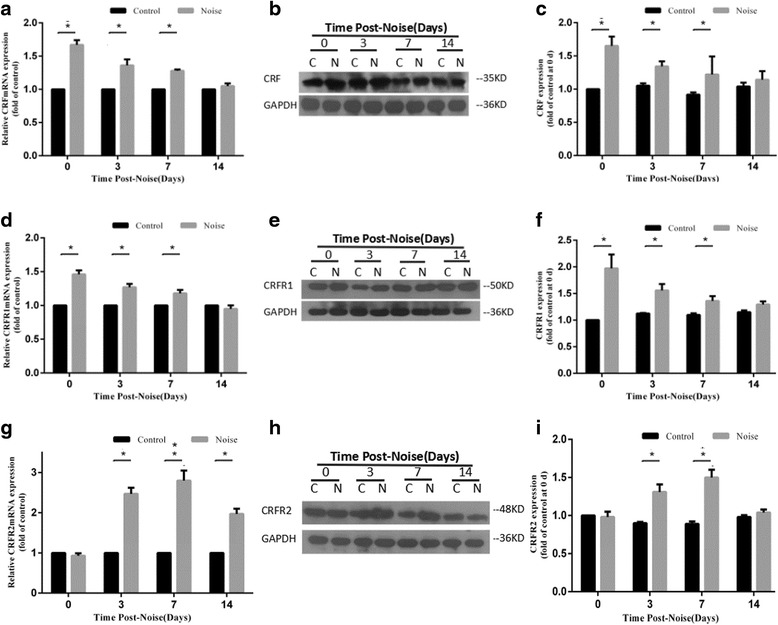
Chronic noise exposure increases the expression of CRF, CRFR1, and CRFR2 in the hippocampus. Comparison of *CRF*, *CRFR1*, and *CRFR2* mRNA expression levels in control and noise-exposed rats by quantitative real-time PCR at various time points following the cessation of noise exposure (**a**, **d**, **g**). Western blot analysis of hippocampal CRF, CRFR1, and CRFR2 expression under C (control) and N (chronic noise exposure) conditions (**b**, **e**, **h**). GAPDH was used as a loading control. Quantification of immunoreactive band density measured in panels **b**, **e**, and **h** was normalized against GAPDH (**c**, **f**, **i**). Data are presented as the percentage changes relative to control samples. Bars represent means ± SD *p < 0.05, compared with respective controls (*n* = 6 per group)

To assess the influence of chronic noise exposure on the CRF system, we examined the expression levels of CRF, CRFR1, and CRFR2 by using RT-PCR and Western blotting, respectively. Results showed that the expression levels of CRF and CRFR1 mRNA and protein in hippocampus were significantly increased after exposure to noise for 30 days, with an increasing trend that persisted for up to 7 days after the final noise exposure (Fig. 2a–f). Meanwhile, a delayed increase of CRFR2 in mRNA and protein levels was also observed at days 3 and 7 after noise exposure (Fig. 2g–i).

### Chronic noise exposure causes tau hyperphosphorylation in the hippocampus

**Fig. 3:**
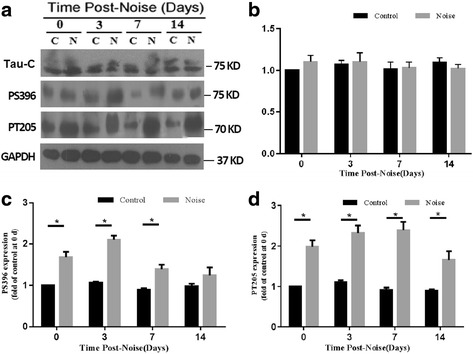
Chronic noise exposure increases hyperphosphorylated tau in the hippocampus. Western blot analysis of hippocampal phosphorylated tau under control (C) and chronic noise exposure (N) conditions. Immunoblot panels were probed with anti-tau or phosphorylation-dependent anti-tau antibodies as indicated (**a**). The density of the immunoreactive bands was quantified and is presented as the percentage change relative to the control samples (**b**, **c**, and **d**). GAPDH was used as the loading control. Bars represent mean ± SD (*n* = 6 for each condition). *p < 0.05, compared with respective controls (*n* = 6 per group)

The levels of total tau and p-tau in the hippocampus were evaluated with three antibodies, which are specific to C-terminal epitope of tau independent of its phosphorylation and different phosphorylation-dependent epitopes of tau at Thr 205 and Ser396, respectively, by Western blotting of the hippocampal extracts in at various times after the end of the noise exposure. Immunoblot analysis showed that 30-day noise exposure results in constant tau hyperphosphorylation at p-T205 and p-S396 at days 0, 3, and 7 after exposure in hippocampus, while no difference was found in total tau protein level between the noise-exposed groups and their respective control groups up to 14 days after noise exposure (Fig. 3a–d).

### Chronic noise exposure causes co-localization of CRF and p-tau in the hippocampus

**Fig. 4:**
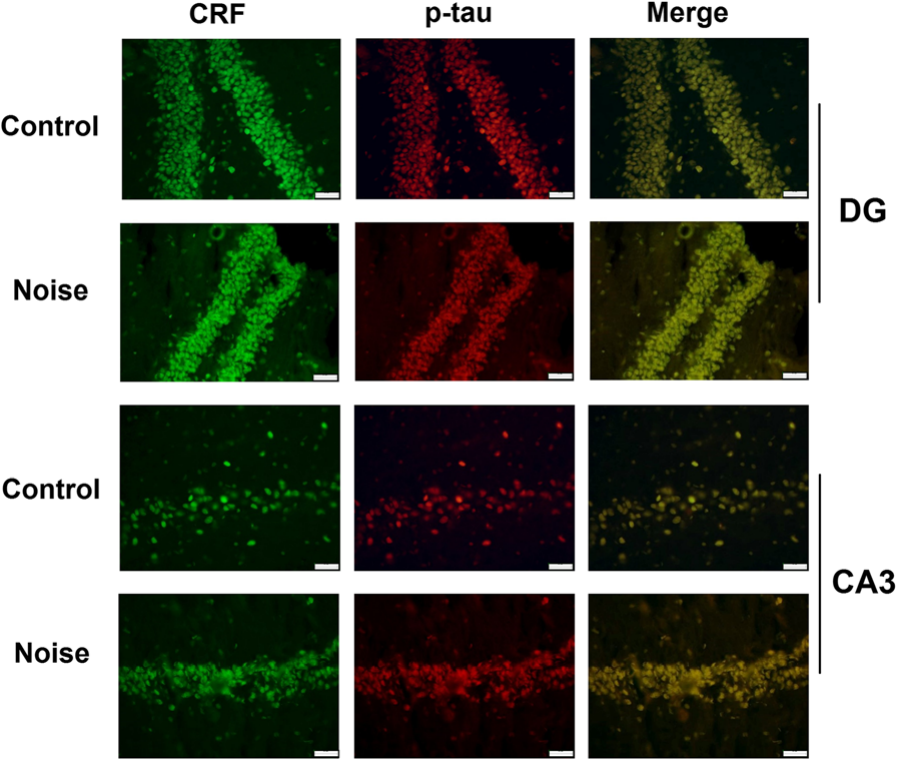
Co-localization of CRF and p-tau (T205) in the rat hippocampus. CRF fluorescence (green), p-tau fluorescence (red), and merged images of CRF and p-tau (yellow) in the hippocampus. Representative images of the rat hippocampal DG region and CA3 region immediately after cessation of noise exposure. Scale bar = 20 μm

To evaluate the association between CRF and p-tau, double labeling of CRF and p-tau was performed on sections of the rat hippocampus. A high proportion of CRF-positive cells co-localized with p-tau in the DG and CA3 regions of the hippocampus. CRF immunoreactivity within cell bodies showed green cytoplasmic staining, and positive p-tau staining was also observed in the cytoplasm while yellow is the double staining of CRF and p-tau (Fig. 4).

## Discussion

In this study, we evaluated the aftereffects of chronic noise stress on the expression of the CRF system in the hippocampus and explored its relationship to tau hyperphosphorylation. The results presented here provide novel insight into the mechanism underlying this link, which involves noise-induced dysfunction in tau phosphorylation.

Elevated glucocorticoid levels induced by chronic stress are associated with cognitive impairment and AD-like neurodegeneration [14, 15]. A wide variety of studies demonstrates that AD-like hyperphosphorylation of tau protein can be triggered by various environmental insults, such as physical hazards [4, 16–18] and psychological stressors [9, 19], and that this may be mediated by CRFR1 [20]. Previous studies showed that following chronic stress, tau phosphorylation and Aβ accumulation levels in the hippocampus are significantly elevated, and CRF may play a key regulatory role in these changes [10, 12]. Furthermore, the literature suggests that CRF acts via CRFR1 to play a leading role in mediating AD-like changes in selected brain regions although the role of CRFR2 in AD-like changes remains unclear [10]. Our results indicated that chronic noise exposure induces elevated expression of CRF and CRFR1 mRNA and protein in the rat hippocampus; further, we show a delayed increase in CRFR2 mRNA and protein expression continued for at least 7–14 days even after 30 days of noise exposure was terminated, which suggests a cumulative impact of the CRF system on the risk of chronic noise exposure.

The distribution of CRFR2 in the central nervous system is not as widespread as that of CRFR1, and the two receptors seem to perform different functions under physiological conditions [21, 22]. Of relevance to the current study, neurodegenerative diseases often trigger neuroinflammatory changes [23]. CRFR1 has a key role in the regulation of the pro-inflammatory response during neural inflammation in the brain [24, 25], whereas CRFR2 has anti-inflammatory and anti-cytotoxic actions [26]. We have previously demonstrated that long-term noise exposure can lead to inflammatory changes in the hippocampus [5]. The persistent upregulation of CRFR1 implies that CRFR1 may be involved in noise-induced neuroinflammation in the hippocampus. In addition, the receptor subtypes of CRFR1 and CRFR2 have opposing effects, and the opposing regulation of biological responses by multiple CRF receptors may serve to facilitate different coping strategies in response to stress [27]. Therefore, further studies focusing on the availability and functionality of these receptors are required to reveal the mechanisms responsible for the observed AD-like and inflammatory alterations.

We established that chronic noise exposure induces p-tau accumulation in our previous studies [4, 28]. The present study demonstrates that persistent phosphorylation of tau at Ser396 and Thr205 occurs in the hippocampus of rats after chronic noise exposure, which is consistent with similar exposure paradigms in our previous studies [4, 28]. Furthermore, our results show sustained abnormal alterations of CRF and CRFR1 gene and protein expression after long-term noise exposure and co-localization of CRF and p-tau within hippocampal neurons. These data support the existence of interactions between the CRF signaling system and tau phosphorylation. In accordance with the regulatory role of the CRF system on tau phosphorylation during stress [8–10, 12], together with the relationship between CRF signaling and p-tau, we infer that the CRF signaling system is likely to be involved in the process of tau phosphorylation that is induced by chronic noise exposure in the hippocampus.

The major limitation of this study is the absence of exploration of correlation between CRF signaling, tau kinases and phosphatases, and hearing loss in mediating noise-induced tau hyperphosphorylation. The dynamic changes in tau phosphorylation after stress are likely the result of a complex regulatory network of protein kinases and phosphatases [29, 30]. Our previous study showed that glycogen synthase kinase (GSK)-3β and protein phosphatase (PP) 2A might play a causal role in the induction and persistence of tau hyperphosphorylation [4]. Accordingly, there are indications that CRF and GSK-3 mechanisms may be involved in stress-induced tau phosphorylation [31]. According to these findings, aberrant regulation of CRF signaling and tau kinases and phosphatases may lead to prominent changes in tau phosphorylation in the hippocampus after noise exposure. On the other hand, observational study suggests that aging-related hearing loss is associated with accelerated cognitive decline and incident dementia [32]. We have evaluated the noise-induced hearing loss during our previous study, which showed that the threshold of Click-ABR (auditory brainstem response) of rats exposed to 95 dB SPL white noise for 30 consecutive days elevated by about 13.2 dB compared to control (control: 35.67 ± 3.01 dB SPL vs*.* noise-exposed: 48.90 ± 4.53 dB SPL) (data not published). It remains to be determined whether and how noise-induced hearing loss contributes to AD-like tau phosphorylation.

## Conclusion

In summary, our results show that long-term noise exposure causes significant and persistent abnormalities in the CRF signaling system and the hyperphosphorylation of tau in the hippocampus, which is particularly vulnerable to neurodegenerative processes. Furthermore, the co-localization of CRF and p-tau within the neurons indicates that the CRF system is likely to be involved in the process of AD-like changes that occur during noise exposure. However, we acknowledge that the findings in our current and previous studies of noise-induced CRF and p-tau increases do not show a causal link to the p-tau-related pathology in AD. Further detailed studies are required to clarify the molecular mechanisms underlying the regulation of the CRF signaling system in noise-induced AD-like neuropathological changes.

## Abbreviations

AD: Alzheimer’s disease
CRF: Corticotropin-releasing factor
CRFR: CRF receptor
SPL: Sound pressure level
TBST: Tris-buffered saline-Tween

## References

[1] Berglund B, Thomas L, Dietrich HS. Guidelines for community noise: World Health Organization - WHO; 1999. http://apps.who.int/iris/handle/10665/66217.

[2] Cui B, Wu M, She X (2009). Effects of chronic noise exposure on spatial learning and memory of rats in relation to neurotransmitters and NMDAR2B alteration in the hippocampus. J Occup Health.

[3] Sørensen M, Andersen ZJ, Nordsborg RB, Becker T, Tjønneland A, Overvad K (2013). Long-term exposure to road traffic noise and incident diabetes: a cohort study. Environ Health Perspect.

[4] Cui B, Zhu L, She X, Wu M, Ma Q, Wang T (2012). Chronic noise exposure causes persistence of tau hyperphosphorylation and formation of NFT tau in the rat hippocampus and prefrontal cortex. Exp Neurol.

[5] Cui B, Li K, Gai Z, She X, Zhang N, Xu C (2015). Chronic noise exposure acts cumulatively to exacerbate Alzheimer’s disease-like Amyloid-β pathology and neuroinflammation in the rat hippocampus. Sci Rep.

[6] Braak H, Braak E (1991). Alzheimer’s disease affects limbic nuclei of the thalamus. Acta Neuropathol.

[7] Bale TL, Vale WW (2004). CRF and CRF receptors: role in stress responsivity and other behaviors. Annu Rev Pharmacol Toxicol.

[8] Campbell SN, Zhang C, Monte L, Roe AD, Rice KC, Taché Y (2015). Increased tau phosphorylation and aggregation in the hippocampus of mice overexpressing corticotropin-releasing factor. J Alzheimers Dis.

[9] Zhang LF, Shi L, Liu H, Meng FT, Liu YJ, Wu HM (2012). Increased hippocampal tau phosphorylation and axonal mitochondrial transport in a mouse model of chronic stress. Int J Neuropsychopharmacol.

[10] Rissman RA, Staup MA, Lee AR, Justice NJ, Rice KC, Vale W (2012). Corticotropin-releasing factor receptor-dependent effects of repeated stress on tau phosphorylation, solubility, and aggregation. Proc Natl Acad Sci U S A.

[11] Campbell SN, Zhang C, Roe AD, Lee N, Lao KU, Monte L (2015). Impact of CRFR1 ablation on Amyloid-β production and accumulation in a mouse model of Alzheimer’s disease. J Alzheimers Dis.

[12] Justice NJ, Huang L, Tian JB, Cole A, Pruski M, Hunt AJ (2015). Posttraumatic stress disorder-like induction elevates β-amyloid levels, which directly activates corticotropin-releasing factor neurons to exacerbate stress responses. J Neurosci.

[13] Gai Z, Li K, Sun H, She X, Cui B, Wang R (2016). Effects of chronic noise on mRNA and protein expression of CRF family molecules and its relationship with p-tau in the rat prefrontal cortex. J Neurol Sci.

[14] Csernansky JG, Dong H, Fagan AM, Wang L, Xiong C, Holtzman DM (2006). Plasma cortisol and progression of dementia in subjects with Alzheimer-type dementia. Am J Psychiatry.

[15] Lee BK, Glass TA, Wand GS, McAtee MJ, Bandeen-Roche K, Bolla KI (2008). Apolipoprotein e genotype, cortisol, and cognitive function in community-dwelling older adults. Am J Psychiatry.

[16] Feng Q, Cheng B, Yang R, Sun FY, Zhu CQ (2005). Dynamic changes of phosphorylated tau in mouse hippocampus after cold water stress. Neurosci Lett.

[17] Korneyev A, Binder L, Bernardis J (1995). Rapid reversible phosphorylation of rat brain tau proteins in response to cold water stress. Neurosci Lett.

[18] Papasozomenos SC (1996). Heat shock induces rapid dephosphorylation of τ in both female and male rats followed by hyperphosphorylation only in female rats: implications for Alzheimer’s disease. J Neurochem.

[19] Yan J, Sun XB, Wang HQ, Zhao H, Zhao XY, Xu YX (2010). Chronic restraint stress alters the expression and distribution of phosphorylated tau and MAP2 in cortex and hippocampus of rat brain. Brain Res.

[20] Rissman RA, Lee KF, Vale W, Sawchenko PE (2007). Corticotropin-releasing factor receptors differentially regulate stress-induced tau phosphorylation. J Neurosci.

[21] Chalmers DT, Lovenberg TW, De Souza EB (1995). Localization of novel corticotropin-releasing factor receptor (CRF2) mRNA expression to specific subcortical nuclei in rat brain: comparison with CRF1 receptor mRNA expression. J Neurosci.

[22] Farrokhi CB, Tovote P, Blanchard RJ, Blanchard DC, Litvin Y, Spiess J (2007). Cortagine: behavioral and autonomic function of the selective CRF receptor subtype 1 agonist. CNS Drug Rev.

[23] Perry VH, Nicoll JA, Holmes C (2010). Microglia in neurodegenerative disease. Nat Rev Neurol.

[24] Stevens SL, Shaw TE, Dykhuizen E, Lessov NS, Hill JK, Wurst W (2003). Reduced cerebral injury in CRH-R1 deficient mice after focal ischemia: a potential link to microglia and atrocytes that express CRH-R1. J Cereb Blood Flow Metab.

[25] Kritas SK, Saggini A, Cerulli G, Caraffa A, Antinolfi P, Pantalone A (2014). Corticotropin-releasing hormone, microglia and mental disorders. Int J Immunopathol Pharmacol.

[26] Wang MJ, Lin SZ, Kuo JS, Huang HY, Tzeng SF, Liao CH (2007). Urocortin modulates inflammatory response and neurotoxicity induced by microglial activation. J Immunol.

[27] Valentino RJ, Commons KG (2005). Peptides that fine-tune the serotonin system. Neuropeptides.

[28] Cui B, Wu MQ, Zhu LX, She XJ, Ma Q, Liu HT (2013). Effect of chronic noise exposure on expression of N-methyl-D-aspartic acid receptor 2B and tau phosphorylation in hippocampus of rats. Biomed Environ Sci.

[29] Ikedaa Y, Ishiguroa K, Fujita SC (2007). Ether stress-induced Alzheimer-like tau phosphorylation in the normal mouse brain. FEBS Lett.

[30] Okawaa Y, Ishiguroa K, Fujitaa SC (2003). Stress-induced hyperphosphorylation of tau in the mouse brain. FEBS Lett.

[31] Roe AD, Staup MA, Serrats J, Sawchenko PE, Rissman RA. Rissman Lipopolysaccharide-induced tau phosphorylation and kinase activity—modulation, but not mediation, by corticotropin-releasing factor receptors. Eur J Neurosci, 2011,34: 448–456. 10.1111/j.1460-9568.2011.07764.x PMC3148267 21722209

[32] Deal JA, Betz J, Yaffe K, Harris T, Purchase-Helzner E, Satterfield S (2017). Hearing impairment and incident dementia and cognitive decline in older adults: the health ABC study. J Gerontol A Biol Sci Med Sci.

